# London Needs £350,000 Now

**Published:** 1920-06-26

**Authors:** 


### THE BUDGET OF THE VOLUNTARY HOSPITALS.

### London Needs £350,000 Now.

When the private citizen comes to arrange his personal budget in the present times, and ascertains what his income is likely to be in a given period, and in what way he prudently can set aside this sum for one purpose and that sum for another, he has a task which requires considerable thought. But what must be the problem of a large voluntary hospital which, with a reserve fund diminished to vanishing point by a series of lean years, has to look ahead and contrive how best to beg or borrow the necessary funds for increased and increasing expenditure? Such a survey, this year, in nine cases out of ten, makes it clear that income will not meet expenditure, and that the difference between the two sides of the account will be no mere accidental bagatelle, but a sum which will represent debt, and possibly an addition to substantial existing debt. And debt means the payment of interest—every £1,000 of loan means to-day £70 more expenditure in twelve months.

What can a hospital do when things look as bad as they do to-day? Close some wards? This does not help much because the heavy standing charges—rates, water, heating, most salaries and wages—must go on. Economise? But economy has been brought to a fine point already, and, perforce, during the war in many cases the doubtful economy of postponing repairs and repainting has been practised, with the result that there is an accumulation of work, the cost of which will swamp any surplus when the next good year comes.

The only possible policy is to obtain more income, but as most of those who give to hospitals are in difficulty about their personal budgets this is more easily said than done. Yet £350,000 would put the hospitals of London right for the current year. The sum looks large, but it does not represent much more than one shilling a head from each inhabitant of London, and if there ever was an appropriate time for giving that shilling it is Hospital Sunday. Many, perforce, must give less than the shilling, but it is hoped that many will give much more.

On this page will be found a small table of figures familiar to all who have read the Sunday Fund number of The Hospital in past years. It shows the income of the hospitals and dispensaries of the Metropolis under different headings for ten years, including the most recent year in respect of which precise figures can be obtained. Of the £2,376,000 obtained in one year sixty-eight per cent., or £1,602,000, came from the living in the shape of benefactions and payments by patients (including the wounded during the last year of the war). The other thirty-two per cent., or £774,000, may be said to have come from the dead in legacies, and in the shape of interest on investments made in bygone years. But of all this income the only sum that the hospitals can confidently rely upon is the income from investments, amounting at most to under four shillings in the pound of the requirements. This nest-egg is not too large and every effort should be made to keep it intact. To draw upon reserves is, of course, to take the line of least resistance. It would be a thousand pities if the moderate endowment of our hospitals should gradually disappear as a result of frequent drafts for current expenditure. The endowment or reserve fund is all the more important, first, because there is not much likelihood of augmentation from surplus ordinary income; and, secondly, because it is probable that legacies for endowment will not in the near future be as frequent as in the past. Moreover, any legacies or unusual donations which are in the shape of free capital will probably be required to a very large extent for extraordinary expenditure, on necessary extensions, alterations, and improvements, schemes in respect of which are in abeyance.

**METROPOLITAN HOSPITALS' AND DISPENSARIES'**

**Income in the ten years 1909 to 1918.**

| Year | From the Living | | From the Dead | | | | Total Income |
|---|---|---|---|---|---|---|---|
| | Subscriptions, Donations, Patients' Payments, etc. | Percentage of Total | Investments | Percentage of Total | Legacies | Percentage of Total | |
| | £ | | £ | | £ | | £ |
| 1909 | 701,458 | 57 | 294,206 | 24 | 240,523 | 19 | 1,236,187 |
| 1910 | 703,841 | 50 | 309,320 | 22 | 394,478 | 28 | 1,407,639 |
| 1911 | 738,622 | 53 | 329,253 | 24 | 322,949 | 23 | 1,390,824 |
| 1912 | 741,439 | 55 | 338,425 | 25 | 272,090 | 20 | 1,351,954 |
| 1913 | 845,908 | 52 | 349,295 | 22 | 424,339 | 26 | 1,619,542 |
| 1914 | 801,239 | 49 | 366,841 | 23 | 451,569 | 28 | 1,619,649 |
| 1915 | 978,294 | 62 | 368,536 | 23 | 242,049 | 15 | 1,588,879 |
| 1916 | 1,143,417 | 63 | 382,377 | 21 | 301,282 | 16 | 1,827,076 |
| 1917 | 1,300,539 | 65 | 414,636 | 21 | 290,025 | 14 | 2,005,200 |
| 1918 | 1,602,717 | 68 | 430,566 | 18 | 343,274 | 14 | 2,376,557 |

The system of paying patients and of asking patients to contribute a part of the cost of their maintenance is a growing one, and should, without undue pressure upon the patient, in course of time, yield a substantial part of the income. But so many considerations arise, such as the need of additional wards and the position of the medical man, that no uniform plan of action has been yet decided upon by the hospitals.

We hope we have shown that the hospitals this Hospital Sunday need more, and are obliged to ask for more, than they have ever needed or asked before.

We sincerely beg all old supporters to give not less but possibly more than in past years, and we ask those who have never given—and there must be very many potentially charitable people—to give generously, and afford themselves one of the truest delights that life can impart—that of helping the sick poor in their day of need.

